# New Opportunities and Cautionary Insights about Decentralizing and Deglobalizing Clinical Trials During the Great Lockdown

**DOI:** 10.46697/001c.17692

**Published:** 2020-11-03

**Authors:** Denise Dunlap, Roberto S. Santos, David D. McManus, Bryan O. Buchholz, Nathaniel S. Hafer, MaryAnn Picard

**Affiliations:** 1 University of Massachusetts Lowell, USA; 2 University of Massachusetts Medical School, USA; 3 Massachusetts Medical Device Development Center (M2D2), USA

**Keywords:** clinical trials, covid-19, emerging technologies, healthcare, deglobalization, decentralization

## Abstract

The 2020s began with a rare coronavirus (COVID-19) disaster, which led to a pandemic-induced recession and Great Lockdown. In response, there has been a worldwide mobilization of resources to detect, treat, and cure the virus. Some policymakers are advocating for the repatriation of globally distributed healthcare know-how. Without a cure for COVID-19, the ambiguity concerning how coronavirus-related policies will impact international business remains unclear. Through a multi-method approach, our study sheds light on two key healthcare industry trends: decentralization and deglobalization of clinical trials. We offer actionable strategies to not only mitigate these challenges, but also to take advantage of their new opportunities

## INTRODUCTION

Healthcare is a sophisticated and globally integrated industry involving three key activities for the commercialization of new products: R&D, clinical trials, and manufacturing. The bridge between innovation and commercialization is clinical trials. Clinical trials are highly regulated and carefully monitored studies in which volunteers participate in the process to find cures for diseases. Clinical trials take, on average, 6 to 7 years (PhRMA, 2015) with a median cost of $19 million (Moore, Zhang, Anderson, & Alexander, 2018). Given their pivotal role in the commercialization process, this is the focus of our study.

Although conducting multi-country clinical trials is challenging, these trials are critically important to bring safe and effective drugs/treatments to market. In 2019, the global clinical trials market was valued at $46.8 billion with North America accounting for 51.2% of the market (Grand View Research, 2020). Globally, 69% of existing trials and 78% of new trials were suspended/delayed due to the Great Lockdown (Medidata Solutions, 2020). Data show that new patients entering clinical trials from April 2019-2020 were down by 83% in the U.S., 60% in Asia, and 77% in Europe (Medidata Solutions, 2020).

Considering the severe impact that the coronavirus has had on global clinical trials and its gatekeeping role in the discovery of novel diagnostics, treatments, and vaccines, we engaged in a multi-method approach to uncover actionable insights. We developed a database of coronavirus and non-coronavirus-related research activities. We also interviewed companies and industry experts. Both analyses included startups, midsize, and multinational firms. Our quantitative analysis of over 5,000 healthcare companies and qualitative interviews reveals important trends of interest to the international business community, and our new conceptual framework explores whether decentralization and deglobalization clinical trial trends due to the pandemic offer healthcare firms new opportunities, long-term challenges, or both.

## IN INTERNATIONAL BUSINESS, LOCATION MATTERS

Prior research (Porter & Stern, 2001) suggests that location choice is an important consideration for companies to access unique resources, knowledge, and know-how. Kuemmerle (1997) offers two rationales for offshoring business operations: home-base-augmenting and home-base-exploiting. Home-base-augmenting refers to a location in which information flows from the foreign location to the headquarters; a strategy aimed at absorbing local scientific knowledge, while home-base-exploiting refers to a location in which information flows to the foreign location from the headquarters with the intent of efficiently commercializing new products. We view clinical trials as an important home-base-augmenting activity. Foreign clinical trial locations not only represent a means for firms to reduce research costs, but also, and more importantly, an opportunity to recruit a more diverse (e.g., age, race, gender) study population. Any significant disruption to clinical trials could curtail years of planning, recruiting, and monitoring.

## RESEARCH DESIGN

Using both quantitative and qualitative methods, we analyzed over 5,000 healthcare companies and surveyed the 11 Center for Advancing Point of Care Technologies^1^ grant award winners (82% response rate) from the 2019 and 2020 award competitions. These grant awardees represent a diverse population of startups, small, and midsize companies. The surveys were conducted in August 2020 and were followed by telephone interviews. We also interviewed large multinationals and industry experts. These interviews helped to inform our conceptual framework and insights.

## CONCEPTUAL FRAMEWORK

**Figure 1:**
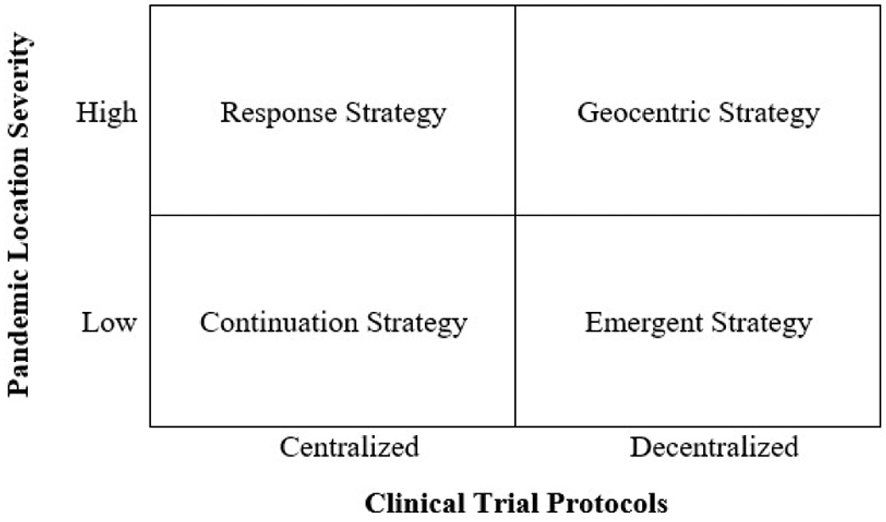
Conceptual Framework

We identified four strategies used by healthcare firms in response to the pandemic based on the severity of its impact and whether the firm used centralized/decentralized protocols. A firm’s ability to utilize a particular strategy was driven by the extent of its global network. These strategies are listed below and illustrated in Figure 1.

- **Continuation Strategy:** Firms face low disruption to local and global clinical trials and reinforce current protocols.
- **Response Strategy:** Local clinical trials are disrupted, but firms leverage their network to continue global clinical trials.
- **Emergent Strategy:** Firms encounter low disruption to local and global clinical trials, but integrate emerging technologies for remote trials.
- **Geocentric Strategy:**^2^ Firms use emerging technologies for remote clinical trials, in addition to leveraging a global network, to overcome local disruptions and enhance global reach.

### CENTRALIZED VS. DECENTRALIZED CLINICAL TRIALS

Clinical trials are governed by centralized protocols. These protocols dictate study participant eligibility, test schedules, procedures, products under investigation, study duration, and desired outcomes. Since the industry relies heavily on volunteer participants, regulatory agencies have enacted strict rules to ensure participant safety is prioritized. A breakdown in oversight could result in patient harm. Thus, clinical trials need to remain centralized to ensure consistency.

To comply with regulatory agencies, firms engaged in clinical trials have institutionalized the centralization of protocols and rigorously oversee their implementation at home and abroad. There is additional scrutiny when conducting multi-country trials because regulations differ considerably across nations. To ensure the highest ethical and procedural standards are met, healthcare firms follow a deliberate home-base-augmenting strategy that limits the autonomy of subsidiaries and external actors to deviate from study protocols. The travel restrictions imposed by the Great Lockdown limited the ability of monitors to audit global trial locations, which resulted in many studies being suspended/delayed.^3^

Given the challenges imposed by the pandemic, firms are looking for ways to minimize disruptions to their research. An approach that has been gaining acceptance is the decentralization of trials. Unlike the traditional clinical trial model, decentralized clinical trials (DCTs) are executed through telemedicine and mobile/local healthcare providers (Clinical Trials Transformation Initiative, 2018). DCTs have been slow to gain traction and represent only 0.5% of all trials, with 63% of these located in North America and 23% located in Europe (Informa, 2020a). Recognizing that there are challenges to the adoption of DCTs, a recent survey found that 78% of clinical trial professionals believed the pandemic was increasing DCT usage (Informa, 2020b). One survey respondent stated, “COVID-19 is placing additional demand for decentralized trials” (Informa, 2020c). In addition, the interviewee noted that there was a greater need for protocol modifications “to allow for more decentralized visits and the need to de-couple some site-based procedures to allow more visits to be remote” (Informa, 2020c). Building on this observation, we found that DCT growth depends on the adoption of digital health technologies, including health information technology, wearables, digital point-of-care technologies, mobile health, telemedicine, and digital records. Our research found that the U.S., U.K., and Germany lead the development of these emerging technologies.

### GLOBALIZING VS. DEGLOBALIZING CLINICAL TRIALS

The extent to which a firm implements centralized versus decentralized clinical trials is motivated by their local versus global presence. Clinical trials have been severely disrupted by the pandemic. Many hospitals have suspended these crucial programs to focus their resources on treating coronavirus patients. The facilities most affected were those located in high outbreak regions. All interviewees said that they experienced significant disruptions. One U.S.-based interviewee noted that all non-coronavirus related clinical trials were suspended and have been slow to restart. Current research supports that patient enrollment for clinical trials has significantly declined with these areas most affected: central nervous system (−76%), respiratory (−86%), endocrine (−88%), dermatology (−91%), and cardiovascular (−95%) (Medidata Solutions, 2020). In addition, one Big Pharma interviewee stated that clinical trials related to elective surgeries (e.g., orthopedics) virtually stopped overnight.

Our data shows that while non-coronavirus-related clinical trials have declined, coronavirus-related clinical trials are growing rapidly, accounting for 20% of all trials. Currently, there are 549 drugs in development with 281 in the U.S., 42 each in China, Canada, and U.K., and 38 in South Korea. We conducted a network analysis to examine the relationship between the drug’s originating country and where its clinical trial was taking place. Network analysis reveals the strength and direction of relationships and identifies the central actors. Of those drugs in development, location data was registered for 420 drugs. We identified countries by their two-character country codes as defined by the U.S. Patent and Trademark Office.

**Figure 2:**
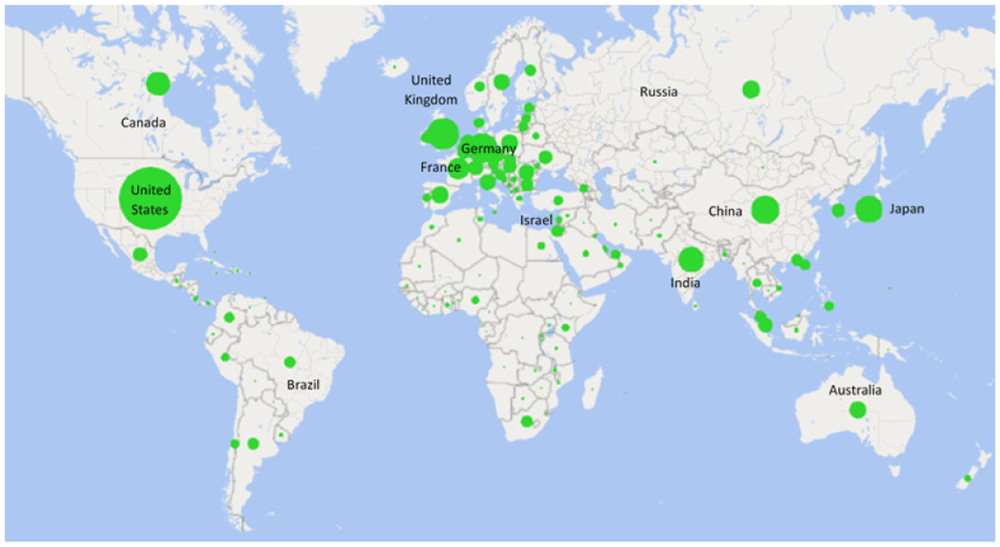
Global Distribution of Clinical Trials Pre-Coronavirus

**Figure 3:**
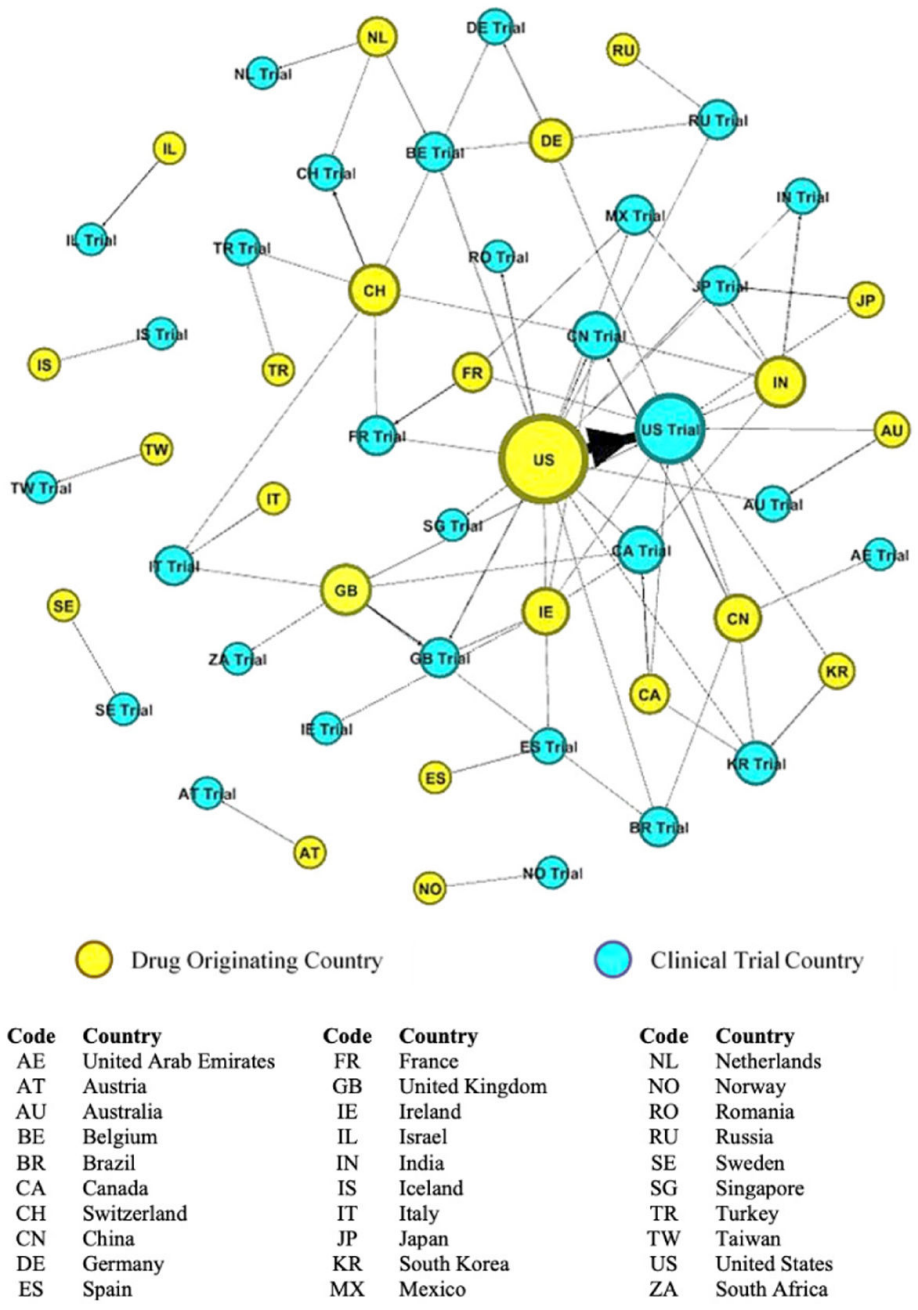
Coronavirus Clinical Trials Network Analysis

Our analysis reveals a far different picture of the diversity customarily found in clinical trials. Prior to the coronavirus outbreak, we found that clinical trials were dispersed across 149 countries (See Figure 2). However, following the outbreak, we found a concerning deglobalization trend. Of the 23 countries developing COVID-19 drugs, clinical trials are being conducted in just 30 countries, with the majority of these being conducted at home rather than abroad (See Figure 3), which raises concerns about the diversity and number of patients needed to develop a worldwide cure. We found that the U.S. occupies the most central position within the network. In this regard, the U.S. conducts more clinical trials at home and conducts the most clinical trials abroad. Other countries have severely curtailed their overseas clinical trials, but those that are conducting trials overseas tend to do so in the U.S. These results reveal some cautionary deglobalization trends that could have long-term consequences. Indeed, to accelerate the development of a cure, firms need to increase recruitment of clinical trial participants from regions that are experiencing larger outbreaks.

We suggest that if deglobalization trends continue, clinical trials could become less representative of the global population, which will introduce increased uncertainty about the safety and efficacy of new treatments/cures. Careful consideration needs to be given to this deglobalization phenomena. Historically, the fastest vaccines developed took 4 years for mumps and, most recently, 5 years for Ebola. Our data on Ebola clinical trials shows that it took a worldwide effort, with over 80 clinical trials occurring in 17 countries for a virus that was predominantly contained in Africa. Unlike Ebola, the coronavirus is felt in every corner of the globe. Currently, there are 195 vaccine trials planned or currently underway. One of our industry experts noted that finding a vaccine requires clinical trials of 30-60 thousand participants each. Further, he said, “it is hard to recruit that many willing volunteers in one country,” which we argue makes the deglobalization of clinical trials an ineffective long-term strategy.

## STRATEGIC LESSONS IN A PANDEMIC

Given the unpredictable nature of the coronavirus, fears of a second wave, and years until a vaccine may be discovered, we identify four strategies that firms can employ to address the impact on their healthcare research. These strategies emphasize the importance of having a global strategy and investing in DCT opportunities.

- **Continuation Strategy:** Despite specific regional shut downs, firms operating in locations that were not severely impacted by the virus maintained their clinical trial operations using centralized protocols. Several of our interviewees remarked that locations outside of mega-cities were becoming more desirable for clinical trials. This strategy involves staying the course and not deviating from pre-existing plans.
- **Response Strategy:** When the severity of the outbreak was high and local operations were shut down, firms with an international presence in lower impacted regions shifted their clinical trials overseas to minimize disruptions. One U.S.-based interviewee, being unable to run clinical trials in Boston, responded to the pandemic by shifting their centralized clinical trials to their Spanish subsidiary. Since Madrid had a lower infection rate than Boston, they were able to begin recruiting participants for their novel oncology clinical trial at a local hospital.
- **Emergent Strategy:** Firms using this strategy were not severely impacted by the virus, but saw the opportunity to use new technologies to decentralize protocols. Several firms we spoke to said that the pandemic was a silver lining in that it increased the value proposition of their DCTs, especially for those receiving regulatory approval prior to the pandemic.
- **Geocentric Strategy:** We found that few firms were able to fully execute this strategy due to international regulations and low DCT adoption. However, areas severely impacted by the coronavirus provided opportunities for enhanced use of these technologies. One firm shared that it was able to modify its testing protocol to monitor patients in hard hit areas via video conferencing during the lockdown. Another firm planned to put DCTs in the hands of trained healthcare workers who could conduct in-home clinical trials in rural populations in India, Asia, and Africa. By thinking locally and acting globally (Perlmutter, 1969), this strategy offers the greatest opportunity to not only increase new patient recruitment, but also minimize research disruption and patient health risk.

## CONCLUSION

Our study highlights that location matters. We offer strategies for how firms can overcome challenges brought on by the pandemic. The importance of maintaining a global presence is even more important during a pandemic because it not only allows for the continuation of clinical trials but also facilitates access to a more heterogenous pool of clinical trial participants. Our network analysis also reveals that greater collaboration among countries is needed to find a cure that is applicable to the global population. DCTs offer an increasingly important avenue to reach a more diverse population. Notwithstanding recent advances in DCTs, there remains a limit to the degree to which clinical trials, a home-base-augmenting strategy, can be fully decentralized (e.g., many clinical trials involve complex treatments that cannot be administered at home). Our conceptual framework shows that firms are better positioned to withstand pandemic-induced disruptions when they draw upon their global networks and incorporate DCTs where appropriate.

## Footnotes

1 CAPCaT is a UMass Medical School/UMass Lowell joint project funded by NIH to advance point-of-care technologies for heart, blood, lung and sleep disorders.

2 This strategy builds upon Perlmutter’s (1969) EPRG framework.

3 Contract research organization IQVIA stated: “80% of its clinical research sites are impacted due to travel restrictions and social distancing measures.” (Informa, 2020b: 12). Our interviews corroborate these findings.
